# The Impact of Macronutrient Intake on Non-alcoholic Fatty Liver Disease (NAFLD): Too Much Fat, Too Much Carbohydrate, or Just Too Many Calories?

**DOI:** 10.3389/fnut.2021.640557

**Published:** 2021-02-16

**Authors:** Theresa Hydes, Uazman Alam, Daniel J. Cuthbertson

**Affiliations:** ^1^Department of Metabolic and Cardiovascular Medicine, Institute of Life Course and Medical Sciences, University of Liverpool, Liverpool, United Kingdom; ^2^Liverpool University Hospitals NHS Foundation Trust, Liverpool, United Kingdom

**Keywords:** non-alcoholic fatty liver disease, carbohydrate, saturated fat, over-feeding, *de novo* lipogenesis (DNL), adipose tissue expandability, fructose, randomized study

## Abstract

Non-alcoholic fatty liver disease (NAFLD) is a growing epidemic, in parallel with the obesity crisis, rapidly becoming one of the commonest causes of chronic liver disease worldwide. Diet and physical activity are important determinants of liver fat accumulation related to insulin resistance, dysfunctional adipose tissue, and secondary impaired lipid storage and/or increased lipolysis. While it is evident that a hypercaloric diet (an overconsumption of calories) promotes liver fat accumulation, it is also clear that the macronutrient composition can modulate this risk. A number of other baseline factors modify the overfeeding response, which may be genetic or environmental. Although it is difficult to disentangle the effects of excess calories vs. specifically the individual effects of excessive carbohydrates and/or fats, isocaloric, and hypercaloric dietary intervention studies have been implemented to provide insight into the effects of different macronutrients, sub-types and their relative balance, on the regulation of liver fat. What has emerged is that different types of fat and carbohydrates differentially influence liver fat accumulation, even when diets are isocaloric. Furthermore, distinct molecular and metabolic pathways mediate the effects of carbohydrates and fat intake on hepatic steatosis. Fat accumulation appears to act through impairments in lipid storage and/or increased lipolysis, whereas carbohydrate consumption has been shown to promote liver fat accumulation through *de novo* lipogenesis. Effects differ dependent upon carbohydrate and fat type. Saturated fat and fructose induce the greatest increase in intrahepatic triglycerides (IHTG), insulin resistance, and harmful ceramides compared with unsaturated fats, which have been found to be protective. Decreased intake of saturated fats and avoidance of added sugars are therefore the two most important dietary interventions that can lead to a reduction in IHTG and potentially the associated risk of developing type 2 diabetes. A healthy and balanced diet and regular physical activity must remain the cornerstones of effective lifestyle intervention to prevent the development and progression of NAFLD. Considering the sub-type of each macronutrient, in addition to the quantity, are critical determinants of liver health.

## Introduction

There have been many feeding/overfeeding studies performed and published that have elegantly outlined the whole body, multi-organ, and molecular/cellular effects of human overfeeding in an effort to recapitulate the chronic nutrient excess that has characterized the current obesity epidemic, providing mechanistic insight into the biological adaptations that occur with weight gain and development of overweight/obesity. These studies have been undertaken in individuals with an array of differing baseline characteristics [age, body mass index (BMI), insulin sensitivity, metabolic health status, obesity prone/resistant genotype etc.] and have adopted different study designs (parallel arm vs. cross-over), of progressively longer duration (from hours to many months). They have imposed different feeding regimes (caloric quantity vs. different macronutrient compositions [fat vs. carbohydrate etc.]) with the aim of weight maintenance, or of inducing identical *absolute* or *relative* weight gain for a fixed time period, some with concomitant changes in physical activity. The focus of these studies has been hugely varied examining the effects on energy balance, on structure and function of specific organs/tissues (adipose tissue, skeletal muscle, liver, brain, pancreas) and on inter-organ cross talk. Various experimental techniques have been employed including assessment of behavioral responses, dynamic metabolic assessment using an array of indirect calorimetry, metabolic chambers, stable isotopes, hyperinsulinaemic-euglycaemic clamps, and meal tolerance tests, imaging with dual energy x-ray absorptiometry (DEXA)/computerized tomography (CT)/magnetic resonance imaging (MRI) to assess changes in regional and total body composition and tissue (adipose tissue/skeletal muscle) biopsies, often used in parallel to provide complementary data and an integrated perspective. More than 300 such studies have been recently comprehensively reviewed in an extensive systematic review of human overfeeding studies (1).

The purpose of this narrative review is to examine the impact of different dietary regimes, frequently with contrasting macronutrient composition, seeking evidence from recent overfeeding studies that have provided valuable mechanistic insight, to examine factors that drive liver fat accumulation and damage, in an attempt to better understand the pathophysiology of non-alcoholic fatty liver disease (NAFLD). It is not intended to provide exhaustive epidemiological data nor review evidence for specific dietary manipulations or physical activity interventions, despite a clear acknowledgment that cardiorespiratory fitness, regular physical activity, and 5–10% weight reduction remain key cornerstones of ideal management (2, 3).

## Epidemiology of NAFLD

NAFLD is a major public health problem ranging from hepatic steatosis, an excessive accumulation of intrahepatic triglycerides (IHTG) affecting approximately a quarter of adults, to non-alcoholic steatohepatitis (NASH), in which hepatic inflammation and cellular injury occurs leading eventually to fibrosis, the key driver of cirrhosis (4, 5). Hepatocellular carcinoma (HCC) has an annual incidence of around 10 per 1,000 person years in NAFLD cirrhosis, although NASH has been found to be associated with an elevated risk of HCC even in the absence of cirrhosis (6).

Hepatic fat content is an important driver of the metabolic syndrome, partly related to increased hepatic insulin resistance (7), and therefore is associated with obesity and related metabolic disorders, namely insulin resistance, prediabetes, type 2 diabetes (T2D), and cardiovascular disease (8, 9). NAFLD is also associated with a number of other systemic complications including chronic kidney disease, and a variety of malignancies, hepatic, and extra-hepatic (10).

NAFLD was not recognized as a clinical entity until 1980 (11), but has been exponentially increasing in prevalence in all populations across all ages (including pediatric and adolescent populations), socioeconomic groups (disproportionately afflicting the more socioeconomically deprived), and in developing countries where Western diets have become more common place (4).

Both over nutrition and sedentary lifestyle has been demonstrated to be associated with the NAFLD spectrum in both animals and humans (12–14), and thus improved nutrition and increased physical activity serves as a major therapeutic route to prevention and treatment. Dietary composition has an important impact on the pathogenesis of NAFLD and different dietary macronutrient composition influences the pathways, mediators, and magnitude of weight gain-induced changes in IHTG content. Excessive consumption of fat and sugars are the root causes of human metabolic disease with saturated fat and fructose being the main dietary components that stimulate hepatic lipid accumulation and progression to NASH, whereas unsaturated fat, choline, antioxidants, and high protein diets appear to play a protective role. The European Association for the Study of the Liver support “exclusion of processed food, and food and beverages high in added fructose,” as well as a macronutrient composition in line with a Mediterranean diet however, these recommendations are only supported by evidence graded “moderate” in quality (2), and other organizations including the American Association for the Study of Liver Disease make no dietary recommendations for individuals with NAFLD (15). The evidence around these dietary components will be discussed in more detail.

## Nutrient-Induced Drivers of Liver Fat Accumulation

### Distribution of Excess Energy Amongst Tissues

**Figure 1:**
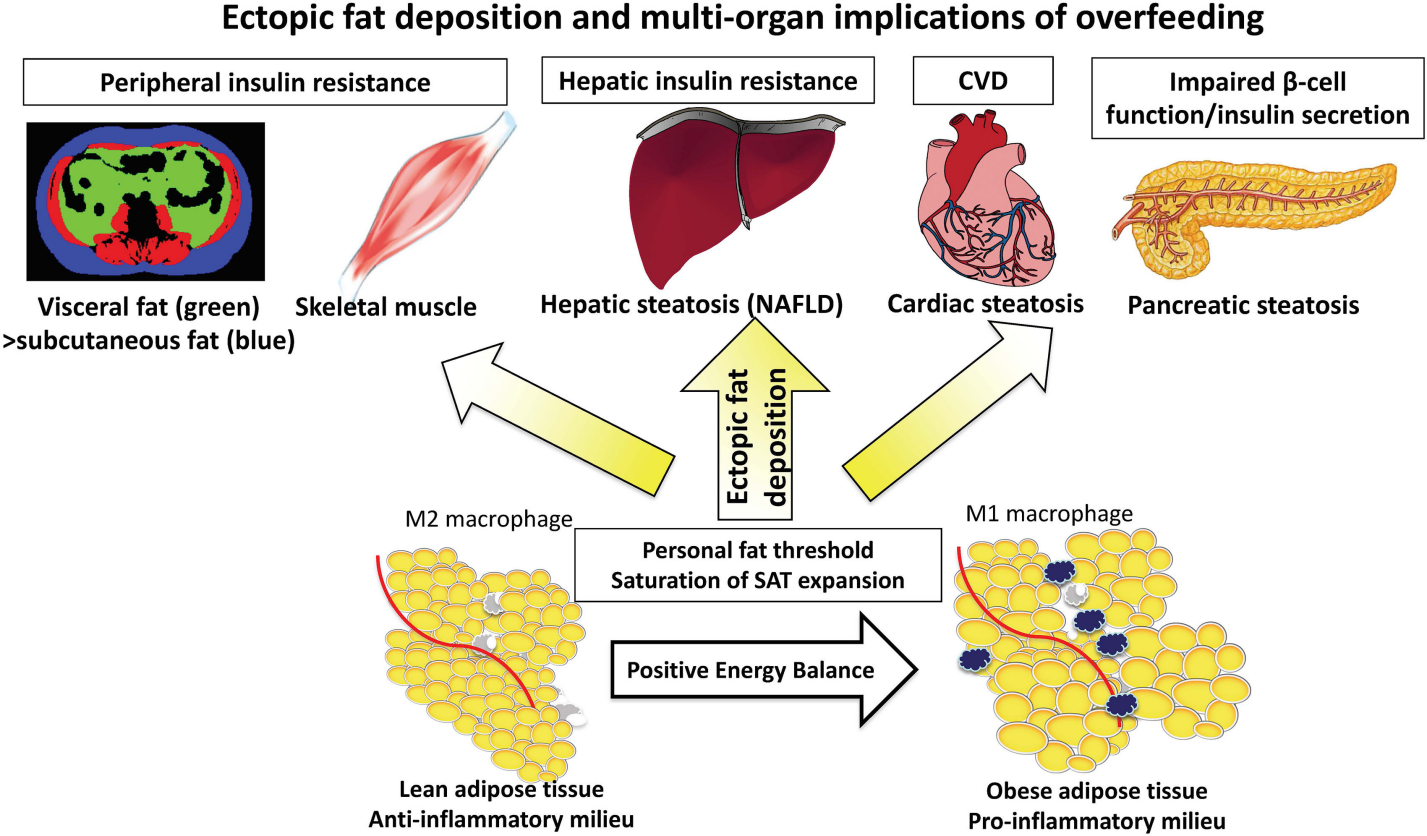
The “adipose tissue expandability” hypothesis leading to deposition of ectopic fat into the liver and other organs.

Although excessive energy intake is a key driver of NAFLD, relatively few human studies have investigated the influence of (isocaloric) dietary composition on biological processes occurring in the liver and ectopic fat accumulation to understand the metabolic consequences of contrasting dietary fats and carbohydrates. Important inter-organ crosstalk occurs between the gut (small intestine), liver, and other peripheral organs including adipose tissue and skeletal muscle (Figure 1).

### Biological Mechanisms to Explain Metabolic Phenotypes

Results from transgenic animal studies, in which subcutaneous adipose tissue (SAT) may undergo massive expansion, demonstrate SAT to be metabolically inert, providing a safe haven for toxic lipids, with consequently reduced ectopic fat (e.g., liver/pancreas/visceral fat) and preservation of insulin sensitivity (16). In contrast, a lesser capacity for SAT expansion is associated with greater ectopic fat deposition, development of systemic insulin resistance, metabolic syndrome, and atherosclerosis (17).

### The Adipose Tissue Expandability Hypothesis

The *adipose tissue expandability* hypothesis has been proposed suggesting that SAT expansion occurs during positive energy balance, but that once the SAT capacity to store energy has been exceeded and maximal SAT expansion has occurred, there is widespread organ-specific ectopic fat deposition (steatosis) in visceral adipose tissue (VAT), liver, pancreas, cardiac, and skeletal muscle (Figure 1) (18). The functional consequences on the organs is lipotoxicity causing hepatic insulin resistance and impaired beta cell function (a sequence of events explained by the *twin cycle hypothesis*) in addition to myocardial dysfunction (19). The absolute storage capacity of SAT has a huge inter-individual variation, unique to the individual as proposed by the *personal fat threshold* (18).

### Regulation of Liver Fat Content

**Figure 2:**
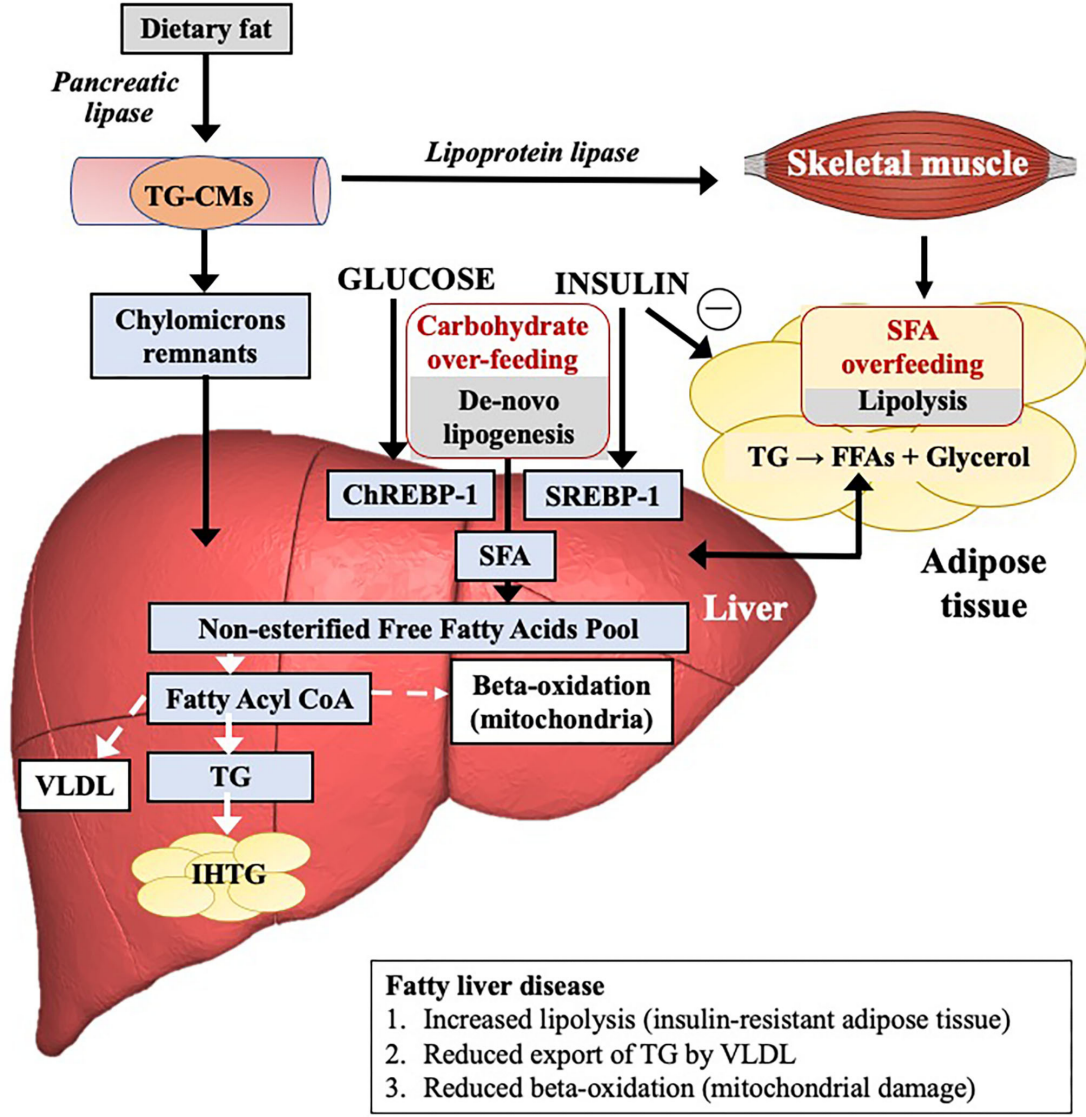
The mechanistic pathways leading to the accumulation of intrahepatic triglycerides and influence of fat or carbohydrate overfeeding on these pathways.

The quantity of IHTG is dependent upon the relative balance of lipid inflow and lipid removal (Figure 2). Lipid influx maybe derived from a variety of metabolic sources including dietary intake/intestinal (15%), adipose tissue lipolysis (increased flux of non-esterified fatty acids, NEFAs) (60–80%) and hepatic *de novo* lipogenesis (DNL) (endogenous synthesis of saturated fatty acids (SFAs) including palmitate from glucose, fructose, or both) (5%). All contribute to liver fat accumulation in distinct proportions. Lipid removal is mediated by both mitochondrial fatty acid β-oxidation and re-esterification to triglyceride (TG) which is either stored or exported into the systemic circulation as very low-density lipoprotein (VLDL). Liver fat accumulates differentially according to the fatty acid composition and/or carbohydrate content/type but significantly appears to do so through different molecular/cellular pathways. Increasing levels of IHTG can drive insulin resistance which in turn may increase the rate of DNL (20). Insulin-resistant adipose tissue also leads to enhanced lipolysis.

### Molecular Pathways in the Liver

Different dietary patterns or certain nutrients may directly promote NAFLD by promoting hepatic TG accumulation and inhibiting antioxidant activity, and indirectly by affecting insulin sensitivity and post-prandial TG metabolism. In general, these include simple sugars (fructose), SFAs, trans-fatty acids and animal protein. Nutrients may do so by acting on a variety of hepatic nuclear receptors to regulate these processes including the liver X receptor (hepatic fatty acid synthesis), the farnesoid X receptor (VLDL assembly), and the peroxisome proliferator-activated receptors (PPARs: PPAR-α, fatty acidv oxidation; PPAR-γ, anti-inflammatory function; PPAR-δ suppresses hepatic lipogenesis, and reduces the hepatic expression of pro-inflammatory genes), as well as cytoplasmic transcription factors such as sterol regulatory element-binding protein (SREBP)-1.

### Contribution of Glucose-Dependent Insulinotropic Polypeptide (GIP) Release and the Gut Microbiome to NAFLD

The gut may modulate fat accretion through different patterns of secretion of intestinal GIP (being most potently stimulated by saturated fat) (21). Additionally, the gut plays a role in NAFLD progression with alterations in gut permeability, the microbiome, and associated endotoxemia contributing to the risk of NAFLD and NASH. While the overall community structure of the gut microbiota appears to remain resilient to short-term overfeeding with both fats and simple sugars, carriage of the anaerobe Bilophila has been identified as a potential risk factor for diet-induced liver steatosis (22). There is also evidence that certain patterns of microbiota (e.g., in T2D) may produce metabolites, specifically histidine-derived imidazole propionate, that can induce insulin resistance by impairing signal transduction through the insulin signaling pathway. These data provide an important mechanistic link between altered patterns of gut microbiota and whole-body metabolism (23).

### Timing and Pattern of Ingestion

Although beyond the scope of this review, the timing of macronutrient intake relative to sleep/wake cycle and the pattern of ingestion is also relevant (24, 25).

## Macronutrient Composition

**Figure 3:**
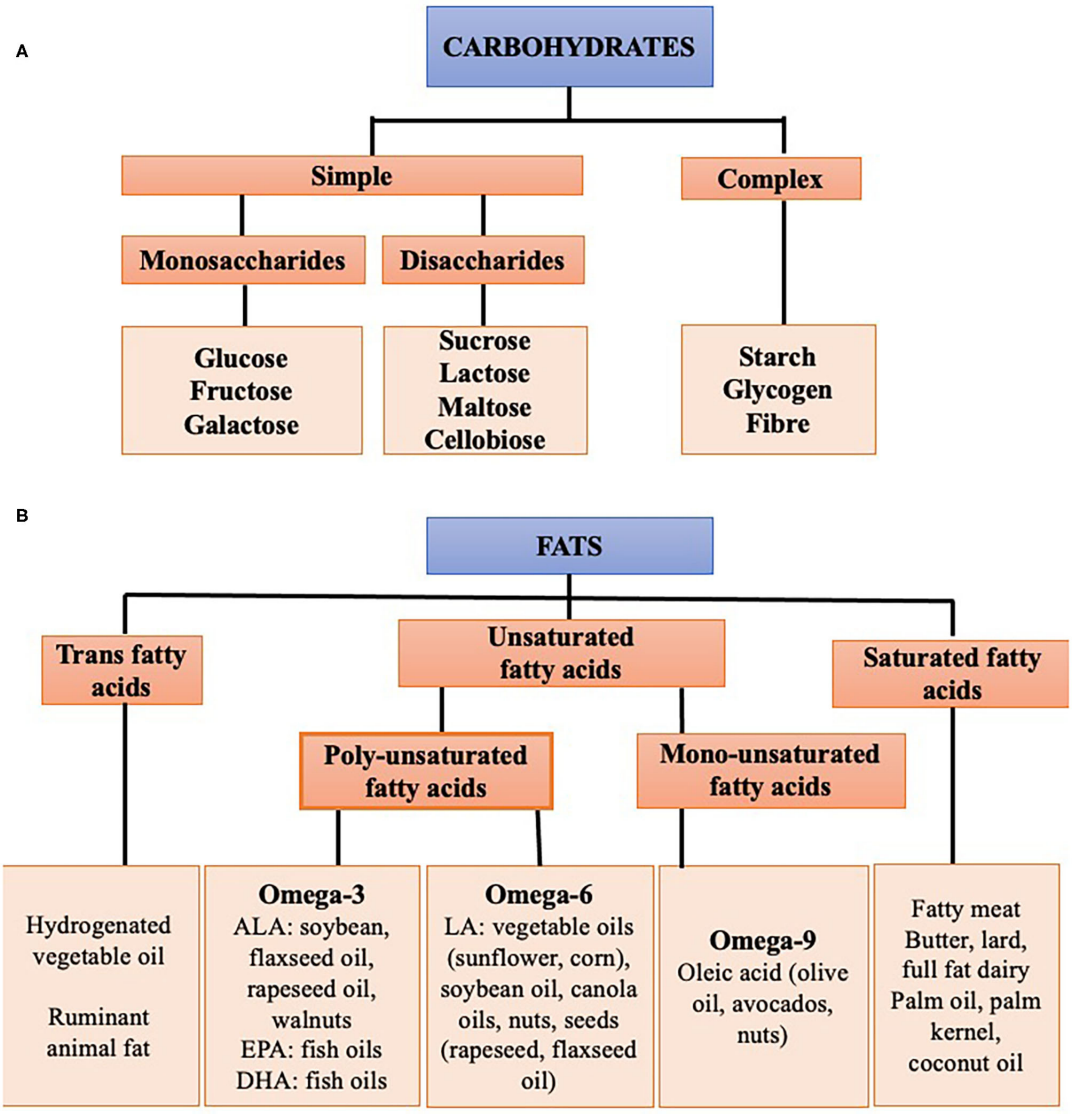
Subtypes of macronutrients **(A)** carbohydrates, **(B)** fat.

Before discussing the effects of overconsumption, it is useful to summarize the different types and subtypes of macronutrients (Figure 3).

### Carbohydrates

Carbohydrates are the sugars, starches and fibers found in fruits, grains, vegetables, and dairy products. They can be divided into whole vs. refined carbohydrates. Whole carbohydrates include fruits, leafy greens, starchy vegetables, beans, peas, lentils, and whole grains. These carbohydrates are high in fiber, vitamins, minerals, antioxidants, and water and are minimally processed. Refined carbohydrates are processed to remove the protein and fat-rich germ and fiber-rich bran, leaving only the starchy endosperm. They are therefore low in fiber and micronutrients. Examples include white rice, white bread, pastries, sugary cereals, sugary drinks, and sweets.

#### Fructose

Fructose is a monosaccharide commonly found in fruits, vegetables and honey (5–10% fructose), but due to its sweetness is also a major component in the two most commonly used sweeteners, sucrose, and high fructose corn syrup (HFCS, a mixture of fructose and glucose monosaccharides). HFCS is found in soft drinks and pre-packaged foods.

#### Glucose

Glucose is present in all major carbohydrates, such as starch and table sugar. Glucose is metabolized primarily by glucokinase or hexokinase. It is found in its free state in fruits and plants and is a component of fruit juices, sugar-sweetened beverages (SSB), and processed foods.

#### Sucrose

Sucrose is a disaccharide composed of glucose and fructose. It occurs naturally in sugarcane, sugar beets, dates, and honey. It is often the sole component of table sugar and is also found in high quantities in maple syrup and chocolate.

#### Lactose

Lactose is composed of glucose and galactose and is found predominantly in milk and other dairy products.

### Fats

Fats can be divided into three groups: trans-fatty acids, saturated fat and unsaturated fat.

#### Trans-Fatty Acids (TFAs)

Trans-fatty acids (TFAs) are found in fast and fried foods, partially hydrogenated vegetable oil, cakes and pastries, and can increase serum cholesterol. The availability of industrial TFA from partially hydrogenated vegetable oils is being increasingly limited and is forbidden in some countries. Small amounts of TFAs are also found in fats from ruminant animals.

#### Saturated Fatty Acids

Saturated fatty acids generally originate from animal sources including fatty meat, butter, full fat dairy products, and tropical oils, including palm oil and coconut oil. Excessive consumption of SFAs, common in western diets, can cause insulin resistance and raise serum low-density lipoprotein (LDL) cholesterol levels.

#### Unsaturated Fatty Acids

Unsaturated fatty acids are either poly or monounsaturated. Sources of monounsaturated fats (MUFAs) include olive oil, rapeseed oil, soy, avocados, and certain nuts. Monounsaturated fats reduce levels of LDL cholesterol while maintaining high concentrations of high-density lipoprotein (HDL) cholesterol. Polyunsaturated fats (PUFAs) can be divided into the omega-3 (n-3) and omega-6 (n-6) families. Omega-6 fats are found in vegetable oils (corn, sunflower oil), seeds (rapeseed, flaxseed oil), soybean oil, and nuts. Omega-3 fats are sourced from fish oils and algae, as well as rapeseed oil, soy, walnuts and flaxseed oil. PUFAs have been shown to improve LDL cholesterol levels to an even greater extent than MUFAs, and importantly may also improve insulin sensitivity (26).

## Observational Data of Dietary Patterns

Epidemiological data on dietary risk factors for NAFLD from population-based studies are scarce. Most human studies are observational and retrospective, allowing limited inference about causal associations. From those studies that are available we have seen clear positive associations of NAFLD with intake of red and processed red meat, poultry and cholesterol and negative associations with dietary fiber intake (27, 28). Lower hepatic PUFA levels were also associated with NAFLD in a cross-sectional study (29).

## Effect of Excess Energy Intake and Specific Macronutrients on Liver Fat

Experimental overfeeding can be implemented using excess energy from any form of hypercaloric diet. The diet may be balanced or unbalanced according to the relative amounts of fat, carbohydrate or protein, or may involve consumption of a particular type of fat or carbohydrate. The mechanisms by which specific macronutrients in excess cause a change in liver fat (and indeed other ectopic fat depots) are of interest. The methodologies range from more simple study designs to complex multi-modality assessments concomitantly examining changes in functional adipose tissue characteristics, body composition (with DEXA and/or MRI/^1^H-magnestic resonance spectroscopy, MRS) and the metabolic consequences (using oral glucose tolerance test or euglycaemic clamps) (30). Key overfeeding studies that have provided an insight into the impact of short- and longer-term excess carbohydrate or fat ingestion on liver fat content are shown in Tables 1–3; and their effect on adipose tissue expansion, insulin sensitivity, and other metabolic profiles are shown in Supplementary Tables 1–3.

**Table 1:** Summary of studies examining the impact of carbohydrate over-feeding on liver fat.

**References**	**Participants**	**Design**	**Intervention**	**Duration**	**Body weight (kg)**	**Liver fat (^1^H-MRS, %)**	**ALT, AST (U/L)**	**Markers of DNL**
**Shorter duration (≤1 week)**								
Lê et al. (31)	Off-spring of people with T2D (OffT2D) (*n =* 16) Controls (*n =* 8)	Randomized Cross-over	Isocaloric diet + 3.5 g/kg/FFM fructose + 35% EE (HFrD)	7 days 4–5 week washout	Controls +0.6 OffT2D +1.0 Diet, *p <* 0.05 Groups, *p =* ns	OffT2D +79% Controls +76% Groups, *p =* ns	*ALT:* Controls 16.9 ± 1.2 → 24.9 ± 4.2; OffT2D 16.4 ± 1.0 → 29.3 ± 4.2 Groups, *p =* ns	*18:2n26: 16:0 in serum VLDL TAG*: Controls 0.36 → 0.31 (*p <* 0.001) OffT2D 0.35 → 0.27 (*p <* 0.001) Groups, *p =* ns
Ngo Sock et al. (32)	Non-diabetic men (*n =* 11)	Randomized Cross-over	1. WM+3.5 g/kg/FFM fructose (HFrD) 2. WM+3.5 g/kg/FFM glucose (HGlcD) HFrD/HGlcD + 35% EE	7 days 2–3 week washout	HFrD +0.6 HGIcD +1.0 HFrD vs. WM, *p <* 0.01 HGIcD vs. WM, *p <* 0.05	HFrD +52 ± 13%, HGIcD +58 ± 23% HFrD vs. WM, *p <* 0.05 HGIcD vs. WM, *p =* ns HFrD vs. HGIcD, *p =* ns	*ALT, AST: p =* ns vs. WM diet	
Theytaz et al. (33)	Non-diabetic men (*n =* 9)	Randomized Cross-over	1. HFrD (3 g/kg) + placebo (+36% EE) 2. HFrD (3 g/kg) + EAA (HFrAA) (+38% EE)	6 days 4–10 week washout	*p =* ns with diet	WM +1.27 ± 0.31 HFrD +2.74 ± 0.55 (*p <* 0.05 vs. WM) HFrD vs. HFrAA, *p <* 0.05		*^13^C palmitate production (nmol/540 min):* WM 1.0 ± 3.9 HFD 182.2 ± 52 (*p <* 0.05 vs. WM)
**Longer duration (> 1 week)**								
Johnston et al. (34)	Non-diabetic men (*n =* 32)	RandomizedCross-overDouble blind	1. Isocaloric HGlD/HFrD 2. Hypercaloric (HclD) (+25% EE) HGlD/HFrD	2 weeks 6 week washout	Results for HclD HFrD +1.0 ± 1.4 (*p <* 0.05) HGlD +0.6 ± 1.0 (*p <* 0.05) HFrD vs. HGlD, *p =* ns	Results for HclD ^a^HFrD +1.70 ± 2.6 (*p <* 0.05) HGlD ‘+2.05 ± 2.9 (*p <* 0.05) HFrD vs. HGlD, *p =* ns	*ALT, AST*: *p =* ns for HclD *p =* ns for HFrD vs. HGlD	
Sevastianova et al. (35)	Non-diabetics genotyped for PNPLA 3. (*n =* 16). Mean BMI 30.6 kg/m^2^	One group	Hypercaloric diet (>1,000 kcal/day simple sugars)	3 weeks	+1.8 ± 0.3 (*p <* 0.0001)	+27% (9.2 ± 1.9 → 11.7 ± 1.9), *p <* 0.05	*ALT:* +28% (50 ± 11 → 64 ± 16), *p <* 0.05 *AST:* +19% (36 ± 4 → 43 ± 6), *p <* 0.05	*16:0: 18:2n26 in serum VLDL TG*: +52% (2.1 ± 0.3 → 3.2 ± 0.5), *p <* 0.05 Positively correlated with liver fat during overfeeding (*p <* 0.05)
Lê et al. (36)	Non-diabetic men (*n =* 7)	One group	Isoenergetic diet + 1.5 g/kg/d fruct (+18%EE)	4 weeks	*p =* ns with diet	*p =* ns		
Silbernagel et al. (37)	Non-diabetic overweight individuals (*n =* 20)	RandomizedParallelParticipants blinded	1. WM + 150 g/d (600 kcal) fructose (HFrd) 2. WM + 150 g/d (600 kcal) glucose (HGld)	4 weeks	HFrD +0.2 ± 0.6 (*p =* ns) HGlD +1.7 ± 0.4 (*p =* 0.001) HFrD vs. HGlD, *p =* ns	HFrdD/HGlD vs. WM, *p =* ns HFrD vs. HGlD, *p =* ns		
Maersk et al. (38)	Non-diabetic individuals (*n =* 47)	RandomizedParallel	Standard diet + 1L/day: 1. Sucrose-sweetened cola (50%E glu/50%E fruct) (430 kcal/d) 2. Aspartame-sweetened diet cola (4 kcal/d) 3. Milk (454 kcal/d) 4. Water (0 kcal/d)	6 months	No comparison with baseline *p =* ns between groups	Cola vs. milk 143%, *p <* 0.05 Cola vs. diet cola 139%, *p <* 0.05 Cola vs. water 132%, *p <* 0.05 ANOVA *p =* 0.01		

*All results show mean ± SEM unless stated otherwise*.

a *Mean ± SD*.

b *Median (interquartile range)*.

1 *H-MRS, magnetic resonance spectroscopy; ALT, alanine transaminase; AST, aspartate aminotransferase; DNL, de novo lipogenesis; T2D, type 2 diabetes; FFM, fat free mass; EE, excess energy; HFrD, high fructose diet; 18:2n26, Linoleate; 16:0, palmitate; VLDL, very low density lipoprotein; TAG, triacylglycerol; WM, weight maintenance diet; HGlcD, high glucose diet; EAA, essential amino acids; PNPLA3, Patatin-like phospholipase domain-containing protein 3; BMI, body mass index; TG, triglycerides; ANOVA, analysis of variance; HclD, hypercaloric diet*.

**Table 2:** Summary of studies examining the impact of fat over-feeding on liver fat.

**References**	**Participants**	**Design**	**Intervention**	**Duration**	**Body weight (kg)**	**Liver fat (^1^H-MRS/MRI %)**	**ALT, AST (U/L)**
**Shorter duration (≤1 week)**							
van der Meer et al. (39)	Non-diabetic men (*n =* 15)	One group	Habitual diet + 800 ml cream/day—high fat high energy (HFHE) diet [added 2,632 kcl/day (94% fat)]	3 days	*p =* ns for BMI (no data on body weight)	^a^+112% (2.01 ± 1.79 → 4.26 ± 2.78), *p =* 0.001	*ALT, AST*: *p =* ns
Wulan et al. (40)	Non-diabetic men 1. South Asian (*n =* 10) 2. White (*n =* 10)	One group	High fat diet (+50% EE: 60%E fat, 25%E CHO, 15%E protein)	4 days		South Asian +33% (*p <* 0.05) White +34% (*p <* 0.05) Ethnicities, *p =* ns	
Wulan et al. (41)	Non-diabetic men 1. South Asian (*n =* 10) 2. White (*n =* 10)	One group	High fat diet (+50% EE: 60%E fat, 25%E CHO, 15%E protein) Subjects stayed in respiration chamber mimicking a sedentary lifestyle.	3 days		South Asian 1.7 ± 1.4 → 2.7 ± 1.9 (*p <* 0.05) White 2.6 ± 3.5 → 3.1 ± 4.9 (*p <* 0.05) Ethnicities, *p =* ns	
**Longer duration (> 1 week)**							
Kechagias et al. (42)	Non-diabetic individuals (*n =* 18)	One group	2x fast-food based meals/day (aim 5–15% weight gain) Baseline (mean): Kcal/day 2,273, CHO 48%E, fat 36%E (38% sat fat) Study (mean): Kcal/day 5,753, CHO 45%E, fat 43%E (43% sat fat)	4 weeks	^a^+6.4 (*p <* 0.001)	^a^1.1 ± 1.9 → 2.8 ± 4.8 (*p =* 0.003)	*ALT:* 22.1 ± 11 → 69.3 ± 76 (*p =* 0.01) *AST*: 28.1 ± 12 → 39.6 ± 23 (*p =* 0.07)
Rietman et al. (43)	Non-diabetic lean individuals (*n =* 29)	Randomized Cross-over	1. High fat diet + normal protein (NP) 2. High fat diet + high protein (HP)	2 weeks	*p =* ns vs. WM *p =* ns NP vs. HP	*p =* ns vs. WM *p =* ns NP vs. HP	
Rosqvist et al. (44)	Non-diabetic normal weight individuals (*n =* 39)	RandomizedParalleldouble-blind, (LIPOGAIN)	Standard diet + 1. Muffins high in SFAs (palm oil) 2. Muffins high in n-6 PUFAs (sunflower oil) Quantity adjusted for 3% weight gain Muffins matched for energy, fat, protein, CHO, cholesterol.	7 weeks	^a^SFA +1.6 ± 0.96 PUFA +1.6 ± 0.85 SFA vs. PUFA, *p =* ns	SFA **+**0.56 ± 1.0 PUFA +0.04 ± 0.24 SFA vs. PUFA, *p =* 0.033	
Johannsen et al. (45)	Non-diabetic individuals (*n =* 29)	One group	+40% EE: 41%E CHO, 44%E fat (40% SFAs), 15%E protein	8 weeks	+7.6 ± 2.1	1.5 ± 0.6 → 2.19 ± 1.0 (*p <* 0.01)	*ALT:* 27.4 ± 12.4 → 38.3 ± 18.9 (*p <* 0.001)
Rosqvist et al. (46)	Non-diabetic overweight individuals (*n =* 60)	RandomizedParalleldouble-blind, (LIPOGAIN-2)	Standard diet + 1. Muffins high in SFAs (palm oil) 2. Muffins high in n-6 PUFAs (sunflower oil) Quantity adjusted for 3% weight gain Muffins matched for energy, fat, protein, CHO, cholesterol	8 weeks	^a^SFA +2.31 ± 1.38 PUFA +2.01 ± 1.90 SFA vs. PUFA, *p =* ns	^a^SFA +53% (**+**1.54 ± 2.0) PUFA−2% (−0.09 ± 1.55) SFA vs. PUFA, *p =* 0.001	*ALT (mkat/L)*: SFA +0.08 ± 0.18, PUFA−0.01 ± 0.14, *p =* 0.035

*All results show mean ± SEM unless stated otherwise*.

a *Mean ± SD*.

b *Median (interquartile range)*.

1 *H-MRS, magnetic resonance spectroscopy; MRI, magnetic resonance imaging; ALT, alanine transaminase; AST, aspartate aminotransferase; BMI, body mass index; EE, excess energy; W, weight maintenance diet; CHO, carbohydrate; SFA, saturated fatty acids; n-6 PUFA, omega 6 polyunsaturated fatty acids*.

**Table 3:** Summary of studies of both fat and carbohydrate over-feeding on liver fat.

**References**	**Participants**	**Design**	**Intervention**	**Duration**	**Body weight (kg)**	**Liver fat (^1^H-MRS/MRI %)**	**ALT, AST (U/L)**
**Shorter duration (≤1 week)**							
Sobrecases et al. (47)	Non-diabetic men (*n =* 37)	RandomizedParallel	1. HFrD (+3.5 g fructose/kg/FFM, +35% energy) 2. High fat diet (Fat) (+30%E as fat) 3. High-fructose high-fat diet (FruFat) (3.5 g fructose/kg/FFM +30%E as fat)	7 days	*p =* ns	HFrD +16% Fat +86% Frufat +133% FruFat vs. HFrD *p <* 0.05 FruFat vs. Fat *p <* 0.05	*ALT*: Only the FruFat diet led to ALT increase
Lecoultre et al. (48)	Non-diabetic men (*n =* 55)	RandomizedParallel	WM diet + either: 1.5/3/4 g fructose/kg/FFM (F1.5, F3, F4) 3 g/kg/day glucose (G3.0) 30%E as SFAs (Fat 30%)	6–7 days	*p =* ns all group vs. controls	^a^*IHCL (mmol/kg):* F3 9.0 ± 8.0 → 18.5 ± 2.5 (*p <* 0.01) F4 13.1 ± 7.9 → 23.7 ± 15.2 (*p <* 0.01) Fat 30% 11.6 ± 8.0 → 21.9 ± 17.2 (*p <* 0.05) F1.5 6.0 ± 3.0 → 5.7 ± 2.5 (*p =* ns) G3.0 12.9 ± 15.0 → 16.1 ± 15.1 (*p =* ns) F3 vs. F1/F4 vs. F1/Glu3.0 vs. F3 all *p <* 0.05	
Surowska et al. (49)	Non-diabetic individuals (*n =* 12)	Randomized Cross-over	Hypercaloric diet (+45% EE) + high in sucrose 1. Low protein, high fat hypercaloric diet (LP-HF): 5%E protein, 25%E fat 2. High protein, low fat hypercaloric diet (HP-LF): 20%E protein, 10%E fat	6 days 4–8 week washout period	LP-HF +0.7 ± 0.1 (*p =* ns) HP-LF +1.4 ± 0.2 (*p <* 0.01)	*IHCL (^1^H-MRS) (mmol/kg ww)*: LP-HF: 25.0 ± 3.6 → 147.1 ± 26.9 HP-LF: 30.3 ± 7.7 → 57.8 ± 14.8 Two-way ANOVA with interaction *p <* 0.001 overfeeding x protein/fat content	
**Longer duration (> 1 week)**							
Luukkonen et al. (50)	Non-diabetic individuals (*n =* 38)	RandomizedParallel	Hypercaloric diet (1,000 excess kcal/day) 1. SAT: Mainly SFAs (76%E SFAs, 21%E MUFAs, 3%E PUFAs) 2. UNSAT: Mainly USFAs (57%E MUFAs, 22%E PUFAs, 21%E SFAs) 3. CARB: 100%E simple sugars	3 weeks	*p =* ns	SAT +55% (4.9 ± 6.6 → 7.6 ± 8.8), *p <* 0.001 UNSAT +15% (4.8 ± 4.9 → 5.5 ± 4.8), *p <* 0.02 CARB +33% (4.3 ± 4.7 → 5.7 ± 5.4), *p <* 0.02 SAT vs. UNSAT, *p <* 0.01 ^b^*Newly synthesized palmitate in VLDL TG (μmol/L):* CARB +33% (96 [47–116] → 190 [61–303]), *p <* 0.05 UNSAT & CARB, *p =* ns	*ALT:* SAT +25% (28 ± 15 → 35 ± 18), *p <* 0.05 UNSAT & CARB, *p =* ns *AST:* SAT +12% (26 ± 5 → 29 ± 6), *p <* 0.05 UNSAT & CARB, *p =* ns
Koopman et al. (25)	Non-diabetic lean men (*n =* 36)	RandomizedParallel	Hypercaloric diet (+40% EE) 1. High fat high sugar + ↑ meal size (HFHS-S) 2. High fat high sugar + ↑ meal frequency (HFHS-F) 3. High sugar + ↑ meal size (HS-S) 4. High sugar + ↑ meal frequency (HS-F) Controls: ad libitum diet	6 weeks	^a^*BMI (kg/m^2^):* HFHS-S +0.6 (*p <* 0.05) HFHS-F +0.9 (*p <* 0.01) HS-S +0.8 (*p <* 0.001) HS-F + 0.5 (*p =* ns) (no data on body weight)	^a^HFHS-F +45% (0.98 ± 0.91 → 1.38 ± 1.26, *p <* 0.05) HS-F +110% (1.49 ± 0.95 → 3.10 ± 2.16, *p <* 0.05) HFHS-F vs. HS-F *p =* ns HFHS-S +19% (0.85 ± 0.32 → 1.05 ± 1.26, *p =* ns) HS-S +14% (0.80 ± 0.45 → 0.93 ± 1.04, *p =* ns)	

*All results show mean ± SEM unless stated otherwise*.

a *Mean ± SD*.

b *Median (interquartile range)*.

1 *H-MRS, magnetic resonance spectroscopy; MRI, magnetic resonance imaging; ALT, alanine transaminase; AST, aspartate aminotransferase; HFrD, high fructose diet; FFM, fat free mass; WM, weight maintenance diet; SFA, saturated fatty acids; IHCL, intrahepatocellular lipids; EE, excess energy; BMI, body mass index; MUFA, monounsaturated fatty acids; PUFA, polyunsaturated fatty acids; VLDL, very low density lipoprotein; TG, triglycerides; ANOVA, analysis of variance*.

Some key findings of these studies have recently been borne out in a systematic review and meta-analysis of 26 randomized controlled trials assessing the effects of dietary macronutrient composition on liver fat content (51). In order to do this only isocaloric diets were considered; a quarter were over-feeding studies, i.e., the dietary intervention was provided as an energy surplus, and the rest involved isoenergetic diets or restricted energy intake compared to baseline. As expected, unsaturated fats were found to reduce liver fat content compared to SFAs (standardized mean difference (SMD) −0.80 [95% CI −1.09 to −0.51). Replacement of carbohydrate with protein also led to a moderate reduction in liver fat overall although only three studies were included (SMD −0.33 [95% CI −0.54 to −0.12]). Finally, a comparison of high-carbohydrate/low-fat *vs*. low-carbohydrate/high-fat diets revealed significant heterogeneity between studies; three studies concluded the high fat diet resulted in lower levels of steatosis, two studies concluded the opposite and seven studies found no difference (overall pooled effect 0.01 [95% CI −0.37 to 0.36]).

### The Impact of Carbohydrate Ingestion and Excess Carbohydrate Consumption on the Liver

A number of studies have examined the relative roles of glucose and fructose in the adverse metabolic alterations associated with excessive sugar consumption. Results from clinical studies indicate that reductions in sugary beverages and total fructose intake, especially from added sugars, have a significant benefit in reducing hepatic fat accumulation (52). This is borne out by the findings from a number of meta-analyses suggesting the consumption of SSBs is related to the risk of metabolic syndrome; increased TG levels, stimulated DNL and increased visceral fat (53, 54). Fructose may drive NAFLD through multiple mechanisms: (i) hepatic: fructose increases the transcription factor carbohydrate-responsive element-binding protein (ChREBP-1), a master regulator of DNL, while it also impairs hepatic β-fatty acid oxidation, (ii) gut-mediated: fructose is principally metabolized by fructokinase which is highly expressed in the small intestine. Metabolism of fructose in the intestine results in disruption of the tight junctions responsible for increased gut permeability, leading to bacterial and bacterial endotoxin translocation (55).

Overfeeding with carbohydrates (simple sugars) has been shown to lead to significant increases in liver fat content in a majority of studies as determined by ^1^H-MRS (Table 1) (25, 31–35, 38, 47, 48, 50). Significant changes in liver fat deposition can occur within a week of overfeeding (31–33, 47, 48) and in some studies this has been shown to be independent of total body weight gain (33, 38, 47, 48, 50). Sevastianova et al. assigned 16 subjects with a mean BMI of 30.6 to a 3 week high carbohydrate diet and reported a 10-fold greater relative change in liver fat (27%) compared to body weight (2%) (35). A randomized study comparing 6 months of overfeeding with sucrose-sweetened cola or milk revealed that individuals consuming the cola had significantly greater liver fat content at the end of the intervention despite energy consumption between the groups being comparable (38).

Various methodologies have demonstrated an increase in hepatic production of the saturated fat palmitate following carbohydrate overfeeding, indicating a link between excess dietary sugar and the accumulation of liver fat through DNL as opposed to lipolysis (Figure 2, Table 1) (31, 33, 35, 50). Furthermore, increases in liver fat following a high carbohydrate diet positively correlate with DNL (35). This suggests that the liver accumulates fat during carbohydrate overfeeding and supports a role for DNL in the pathogenesis of NAFLD.

While the evidence of the relationship between excess intake of mono- and disaccharides with hepatic steatosis is now well-established, studies vary in their findings of whether hepatic transaminases increase following dietary intervention, suggesting there may be an interplay with other risk factors required to bring about liver inflammation (Table 1).

In terms of other metabolic parameters, carbohydrate overfeeding consistently results in raised levels of TG and VLDL-TG, likely a reflection of an increase in DNL, whereas its impact on the rest of the lipid profile is minimal in the majority of studies (Supplementary Table 1). While SAT volumes are generally increased (25, 35, 38), the influence of carbohydrate overfeeding on VAT is particularly pronounced (38). Glycaemic parameters, including fasting plasma glucose and insulin levels, in addition to markers of insulin sensitivity vary excessively between studies with approximately half demonstrating no change following dietary intervention (31–38).

Although some studies have suggested that fructose has more steatogenic potential than glucose, in that it has been demonstrated to lead to enhanced DNL, greater volumes of VAT, altered lipid metabolism and lower levels of insulin sensitivity (56), data from a double blind parallel randomized control trial suggests that the effects on liver enzymes and triacylglcerol (TAG) concentrations are similar (34). Johnston et al. assigned 32 overweight individuals to receive either a high fructose or high glucose hypercaloric diet for 2 weeks with a 6 week washout period (34). The two groups experienced similar increases in weight, and concentrations of TAG in both the liver and serum, in addition to comparable changes in alanine transaminase and aspartate transaminase. No such changes occurred where an isocaloric diet was followed for either group.

The glycaemic index (GI) of carbohydrates does appear to be an important modulator as shown from a crossover trial in which over 80 healthy volunteers consumed either a high or a low GI diet for 7 days (57). The authors reported an increase in the liver fat fraction (and higher hepatic glycogen concentrations) with a high GI diet, whereas liver fat decreased following a low GI diet (57).

Given the association between fructose and NAFLD described above, there may be some confusion regarding fruit which naturally contains fructose. Fruit is part of the Mediterranean diet which is recommended for individuals with NAFLD (2). Most fruits have a relatively low GI depending on factors including ripeness. The quantities of fructose found in fruit are considerably lower than those found in SSBs; for example one pear contains about 12 g fructose, compared to 37 g in a can of cola and ~30 g in a 450 ml bottle of fruit juice (58). A Swedish study recently addressed this issue by randomizing 30 healthy individuals to receive a diet supplemented in either nuts or fruit for 2 months each at +7 kcal/kg body weight per day (59). No change in hepatic fat content was demonstrated in individuals consuming excess fruit despite an almost 3-fold increase in fructose intake.

To summarize there is a clear consensus between studies that overfeeding with simple sugars leads to increased levels of hepatic fat and serum TG levels. While there is some evidence that this effect is independent of total calorie intake and weight gain, additional high quality studies are needed to confirm this. Randomized control trial data suggests that fructose and glucose are equally steatogenic. Multiple mechanisms are at play as described in Figure 2, however DNL appears to be the dominant pathway in the case of carbohydrate overfeeding.

### The Impact of Fat Ingestion and Excess Fat Consumption on the Liver

An increase in dietary fat increases hepatic fat in normal weight and overweight/obese individuals as shown in a number of studies involving either an isocaloric or hypercaloric diet. However, the magnitude and distribution of fat depot expansion varies significantly according to the type of dietary fat consumed (Supplementary Table 2).

#### Isocaloric Diet

In 10 obese women, liver, intra-abdominal, and subcutaneous fat were measured at baseline and after a 2 week isocaloric period consisting of a diet with low vs. high fat, containing either 16%E or 56%E of total energy intake as fat (60). Liver fat decreased by 20 ± 9% and increased by 35 ± 21% with the low- and high fat diet, respectively. Fasting serum insulin showed similar trends decreasing with the low fat diet and increasing with the high fat diet. Intra-abdominal and subcutaneous fat were unchanged (60). Similar findings were found in 20 overweight men allocated to a 3 week low or high fat diet containing either 20%E or 55%E fat with liver fat, decreasing by 13% and increasing by 17% in the low and high fat groups, respectively (61).

The type of dietary fat consumed is relevant. A total of 67 abdominally obese subjects (15% with T2D) were randomly assigned to a 10 week isocaloric diet high in vegetable n-6 PUFAs (PUFA diet) vs. SFA mainly from butter (SFA diet) without modifying the macronutrient intake (62). Body weight slightly increased with no between-group differences however liver fat, measured by ^1^H-MRS, reduced in the PUFA group and increased in the SFA group. No differences were observed for subcutaneous or visceral adipose tissue. Change in liver fat was positively associated with change in serum SFAs. Metabolic profiles including plasma lipid levels were lower during the PUFA diet than during the SFA diet (62).

#### Hypercaloric Diet

LIPOGAIN was a double-blind, parallel group randomized trial examining 39 young, normal weight individuals investigating the importance of dietary fat composition for ectopic fat storage (44). Participants were overfed muffins that were identical in composition except for the type of fat: containing either high levels of SFA (palm oil) or n-6 PUFA (sunflower oil). Participants were provided an additional 750 kcal/day for 7 weeks to their habitual diet to induce identical weight gain (~2.2% increase, 1.6 kg). SFAs markedly increased liver fat and caused a 2-fold larger increase in VAT compared with PUFAs. The increase in liver fat was positive correlated with increases in SFA as measured by plasma palmitic acid. Conversely, PUFAs caused a nearly 3-fold larger increase in lean tissue than SFAs (lean tissue: fat added: 1:1 with PUFAs, 1:4 with SFAs) (44). Although PUFAs, in contrast to SFAs, were noted to be associated with reductions in atherogenic lipoproteins, the weight gain observed with both types of fat overfeeding was associated with hyperinsulinaemia and increased biomarkers of endothelial dysfunction (63).

Recently, Rosqvist et al., conducted a further double-blind randomized trial (LIPOGAIN-2), with a similarly designed intervention comparing overfeeding with SFA from palm oil *vs*. PUFA from sunflower oil (46). Overfeeding was for an 8 week period, followed by 4 weeks of caloric restriction but in an overweight, middle-aged group (mean age 42 years and BMI 28 kg/m^2^) compared with a lean, young population in the earlier study (mean age 27 years and BMI 20, kg/m^2^). The differential effects on liver fat and blood lipid levels were even more distinct. The weight gain observed was 2–2.3 kg and although no differential effect was seen on VAT, pancreatic fat or total body fat, a differential effect was seen on liver enzymes and liver fat, increasing by 50% with SFA but unchanged with PUFA.

#### GIP

The intestinal incretin, GIP seems to play an important role in mediating the impact of saturated fat on the liver. NASH patients exhibit a prolonged elevation of GIP after saturated fat ingestion and this increased GIP response to saturated fat intake is associated with the severity of liver disease (21).

In summary both iso- and hyper-caloric diets have consistently shown that a high fat diet leads to increased levels of hepatic steatosis. Furthermore, randomized control trial data has shown that SFAs lead to significant increases in liver fat, whereas PUFAs are protective in a setting of identical weight gain. This was found to be true for both normal weight and overweight individuals.

### Studies Comparing Carbohydrate and Fat Overfeeding

A small number of randomized studies have compared the impact of fat or carbohydrate overfeeding on liver fat (Table 3). Studies are heterogenous in design, but there is a general trend that overfeeding with SFAs is associated with the greatest risk of hepatic steatosis and increase in transaminases, independent of changes in body weight (47, 50). Luukkonen et al. assigned 38 individuals to receive a hypercaloric diet containing either 100% simple sugars, mainly saturated fat, or mainly unsaturated fat for 3 weeks (50). Lipolysis and DNL were measured under basal conditions and during a euglycaemic hyperinsulinaemic clamp to measure insulin sensitivity. Overfeeding saturated fat increased IHTG more (+55%) than unsaturated fat (+15%, *p* < 0.05), while carbohydrates increased IHTG +33%. Importantly, carbohydrates increased liver fat by stimulating DNL (+98%) while saturated fat did so by significantly increasing the rate of lipolysis (unsaturated fat did not). Additionally, saturated fat induced insulin resistance and endotoxemia and significantly increased multiple plasma ceramides. The diets had also had distinct effects on adipose tissue gene expression (64). Clearly, the metabolic pathways through which different macronutrients increase liver fat are different (50). Finally it is important to note that where overfeeding studies were followed by a hypocaloric diet, changes in weight gain, adverse lipid profiles and liver fat content were all reversed (35, 46).

### Influence of Protein Intake on Risk of NAFLD

The vast majority of epidemiological or mechanistic studies relating to NAFLD have examined carbohydrate and fat intake and/or metabolism but there is emerging evidence that dietary protein intake and specific amino acid patterns is relevant in the pathogenesis of NAFLD (65).

Several overfeeding studies have examined the influence of a hypercaloric diet, with a high protein content, on liver fat accumulation. Bortolotti et al. demonstrated supplementing a high fat diet with protein led to a statistically significant attenuation (~22%) in liver fat accumulation induced by the high fat diet despite the additional energy (66). A further study of a high fat, hypercaloric diet, with a normal vs. high protein intake demonstrated that the higher protein intake tended to lower liver fat, circulating TG concentration and fat mass while increasing fat-free mass (43). A similar finding was observed supplementing a high fructose diet with essential amino acids. This led to a statistically significant attenuation (~16% reduction) in liver fat accumulation when compared with a high fructose diet alone (33). The mechanisms through which protein or amino acid supplementation may attenuate liver fat accumulation are not yet fully understood.

## Mediators of the Overfeeding Response

There are many factors that may influence the response to overfeeding, and thus NAFLD risk, which include the genetic background of the individual (genetic risk of either obesity and/or NAFLD) as well as baseline characteristics including age, sex, BMI, insulin sensitivity, metabolic health status, small for gestational age etc.

### Genetic and Epigenetic Factors May Modulate the Response to Overfeeding

There is evidence that the genetic background of an individual influences the variability of the weight gain and fat storage with caloric excess. This is also likely to apply for liver fat deposition with energy excess. Epidemiological, familial, and twin studies have demonstrated NAFLD has a strong genetic predisposition. Genome-wide association studies led to the identification of the major inherited determinants of hepatic fat accumulation: *patatin-like phospholipase domain-containing (PNPLA3)* I148M gene and *transmembrane 6 superfamily member 2 (TM6SF2)* E167K gene variants (regulating mobilization of TGs from lipid droplets and VLDL secretion, respectively) (67, 68). More recently *membrane bound O-acyltransferase domain-containing 7* (*MBOAT7*), has also been identified (69). These are undoubtedly major determinants of inter-individual differences in liver steatosis, and susceptibility to progressive NASH, yet likely only account for <10% of inherited variability. Their relevance has not yet been assessed in overfeeding studies.

Perhaps more commonly implicated are epigenetic mechanisms, whereby there is interaction between nutrients and the genome by modifications such as DNA methylation, histone modifications, and miRNAs targeting mRNA, that affect gene expression without altering the DNA sequence. These fields of *nutrigenomic* and *nutriepigenomics* are increasingly emerging (70).

### Ethnic-Specific Differences in Response to Overfeeding

Differences in body composition and liver fat deposition are apparent between white Europeans, South Asian and black Africans matched for age, sex, and BMI. South Asian populations are typically characterized by disproportionately more visceral fat accumulation and higher intrahepatic and intramyocellular lipid content than BMI-matched white Europeans), contributing to insulin resistance and higher T2D risk (71–73). In contrast, black African-Caribbean populations consistently have lower levels of liver fat and VAT and higher levels of subcutaneous fat, even after adjustment for BMI or waist circumference (74, 75), although paradoxically despite lower ectopic fat, marked insulin resistance is reported (76). In this population insulin resistance is more strongly associated with abdominal subcutaneous rather than visceral fat (77, 78) and African-Caribbean populations appear more sensitive to the negative effects of ectopic fat, particularly intrapancreatic lipid accumulation (79). Considering South Asians have this lower capacity to store fat in SAT, they may be more susceptible to the negative effects of overfeeding with greater ectopic fat deposition including the liver. When subjected to 4 days of overfeeding (50% excess energy need) with a high fat diet (60%E energy from fat), South Asian and Caucasian men with the same body fat percentage, and similar liver fat content at baseline, showed similar increases in liver fat (33 and 34% respectively) as described in Table 2, Supplementary Table 2 (40). However, the high fat diet had more adverse effects on the lipid profile in the South Asians compared with the Caucasians (41). Longer duration studies in South Asians may reveal different insights while differential effects between overfeeding in Caucasian and African-Caribbean populations are unknown.

### Counteracting the Effects of Overfeeding With Exercise

There is a wealth of epidemiological, cross-sectional and interventional evidence linking physical activity and/or physical inactivity, aerobic capacity, and exercise to liver fat and susceptibility to/protection from NAFLD development and progression. Significantly, exercise can modulate liver fat independent of changes in fat mass (80). Habitual physical activity, cardiorespiratory fitness, and exercise have convincingly been shown to be important in regulating liver fat as shown in a series of cross-sectional and interventional studies (12–14, 81, 82). In a randomized controlled trial of 50 participants, we demonstrated that supervised moderate-intensity exercise, improving cardiorespiratory fitness with small reductions in body weight, led to significantly lower liver fat and improvements in peripheral (but not hepatic) insulin sensitivity (81). These improvements were not sustained following cessation of the exercise supervision (82). Conversely, we demonstrated that 2 weeks of physical inactivity (reducing daily step count from >10,000 to <1,500/day) induced liver fat accumulation and other features of metabolic syndrome (12). Furthermore, we showed that habitual inactivity influences liver fat, with every additional hour of daily sedentary time associated with a 1.15% (95% confidence interval, 1.14-1.50%) higher liver fat content (normal liver fat < 5.56%; NAFLD > 5.56%). (13) Thus, increases or reductions in physical activity are likely to influence the liver's response to nutrient excess/overfeeding. Considering the above evidence, unsurprisingly overfeeding studies have incorporated daily vigorous-intensity exercise into study designs to offset the positive energy balance and counteract the simultaneous imposition of overfeeding with physical inactivity, designed to be representative of modern Western lifestyles (83, 84). Exercise provides this counterbalance at a whole-body and adipose tissue level by preventing the hyperinsulinaemic response and modifying the expression of key adipose tissue metabolic and insulin signaling genes and proteins (83). How exercise may attenuate liver fat accumulation with overfeeding (+/− inactivity) has yet to be examined.

## Conclusions

Dietary intervention studies including overfeeding studies are diverse in their time frames, intervention (different types, subtypes, quantities of macronutrients, isocaloric/hypercaloric), design (parallel/non-parallel studies), control groups, baseline population demographics, and primary outcomes. Some induced weight gain, whilst others did not. Background genetic, ethnicity-dependent, and other baseline factors particularly baseline health and fitness/activity levels will modulate the liver's response to overfeeding. The studies considered here were consistent only in that they all used MRI or MRS to determine changes in liver fat, which is now the gold standard. While this is a narrative review only and no formal evaluation of the quality of evidence was made, we identified consistency among studies reporting an association between increased saturated fat and simple sugar intake and hepatic steatosis. Meta-analysis data suggests that saturated fat can lead to increased liver fat content even in the context of an isocaloric diet. PUFAs have also been consistently shown to have a favorable effect on liver steatosis. It is still unclear whether there is a difference in the ability of fat or carbohydrate to lead to greater levels of IHTGs compared to one another. There is some evidence to support a low GI diet which requires further validation.

There are currently no pharmacological approved agents for NAFLD despite significant investment in this field, and weight loss remains the only proven management for this incredibly prevalent condition. Prevention and disease modification through dietary recommendations, which consider macronutrient intake, have huge potential to be benefit patients. High quality, randomized control studies with adequate baseline controls and longitudinal follow up are essential and urgently needed to provide evidence-based guidance which may to help prevent morbidity and mortality from NAFLD and its associated metabolic conditions.

## Author Contributions

TH, UA, and DC all drafted and reviewed the manuscript and created the figures. TH constructed the tables. All authors contributed to the article and approved the submitted version.

## Conflict of Interest

The authors declare that the research was conducted in the absence of any commercial or financial relationships that could be construed as a potential conflict of interest.
