# Supplementary material for: The Impact of Macronutrient Intake on Non-alcoholic Fatty Liver Disease (NAFLD): Too Much Fat, Too Much Carbohydrate, or Just Too Many Calories?
Source: Front Nutr. 2021 Feb 16;8:640557. doi: 10.3389/fnut.2021.640557 (PMC7921724; doi:10.3389/fnut.2021.640557)
Supplement: Supplementary file 1 [file Table_1.DOCX]

Supplementary table 1. Summary of studies examining the impact of carbohydrate over-feeding on metabolic parameters

| **Ref** | **Participants** | **Design** | **Intervention** | **Duration** | **Body weight (kg)** | **Impact on other metabolic measurements** |
| --- | --- | --- | --- | --- | --- | --- |
| Shorter duration (≤ 1 week) | | | | | | |
| (37) | Off-spring of people with T2D (OffT2D) (n=16)  Controls (n=8) | Randomised  Cross-over | Isocaloric diet + 3.5g/kg/FFM fructose + 35% EE (HFrD) | 7 days  4-5 week washout | Controls +0.6  OffT2D +1.0  Diet, p<0.05  Groups, p=ns | *TAG (mmol/L):* Controls +35%; OffT2D +73% (diet p<0.05, groups p=ns)  *TAG-VLDL (mmol/L):* Controls +51%; OffT2D +110% (diet p<0.05, groups p=ns)*^a^fp-Glucose (mmol/L):* Controls 5.2 → 5.2; OffT2D 5.1 → 5.4 (diet p=ns, groups p<0.05)  *fp-Insulin (pmol/L):* Controls 49 → 57; OffT2D 49 → 60 (diet p<0.05, groups p=ns)  *Lipid oxidation (mg/kg/min):* Controls 0.54 → 0.40; OffT2D 0.48 → 0.39 (diet p<0.05, groups p=ns)  *HISI*: Controls 6.4 → 4.9; OffT2D 6.2 → 4.8 (diet p<0.05, groups p=ns)  *Body fat %:* p=ns for diet & between groups |
| (38) | Non-diabetic men (n=11) | Randomised  Cross-over | 1. WM+3.5g/kg/FFM fructose (HFrD) 2. WM+3.5g/kg/FFM glucose (HGlcD)   HFrD/HGlcD + 35% EE | 7 days  2-3 week washout | HFrD +0.6  HGIcD +1.0  HFrD vs WM, p<0.01  HGIcD vs WM, p<0.05 | *Body fat (%):* WM 15, HFrD 16, HGIcD 16 (HFrD vs WM p<0.05, HGIcD vs WM p<0.05)  *IMCL (mmol/kg/ww)*: HFrD + 49%, HGIcD + 84% (HFrD vs WM p=ns, HGIcD vs WM p<0.05)  *VLDL-TAG (mmol/L):* WM 0.9, HFrD 1.2, HGIcD 1.2 (HFrD vs WM p<0.05, HGIcD vs WM p=ns, HFrD vs HGIcD p=ns)  *LDL, HDL, fp-Glucose, fp-Insulin, HISI*: p=ns vs WM |
| (39) | Non-diabetic men (n=9) | Randomised  Cross-over | 1. HFrD (3g/kg) + placebo (+36% EE) 2. HFrD (3g/kg) + EAA (HFrAA) (+38% EE) | 6 days    4-10 week washout | p=ns with diet | *TG (mmol/L):* WM 0.77, HFrD 1.60 (p<0.05 vs WM)  *VLDL-TG (mmol/L):* WM 0.51, HFrD 1.28 (p<0.05 vs WM)  *fp-Insulin (pmol/L):* WM 73.2, HFrD 105.2 (p<0.05 vs WM)  *Body fat, fp-Glucose:* p=ns |
| Longer duration (> 1 week) | | | | | | |
| (43) | Non-diabetic men (n=32) | Randomised  Cross-over  Double blind | 1. Isocaloric HGlD/HFrD 2. Hypercaloric (HclD) (+25% EE) HGlD/HFrD | 2 weeks  6 week washout | Results for HclD  HFrD +1.0±1.4 (p<0.05)  HGlD +0.6±1.0 (p<0.05)  HFrD vs HGlD, p=ns | *TG (mmol/L):* fructose 0.36 (p=ns), glucose 0.33 (p<0.01); fructose vs glucose, p=ns  *IMCL, fp-Glucose, fp-Insulin, HOMA-IR:* p=ns for hypercaloric diet and difference between fructose/glucose |
| (40) | Non-diabetics genotyped for PNPLA3. (n=16). Mean BMI 30.6 kg/m^2^ | One group | Hypercaloric diet (>1000 kcal/day simple sugars) | 3 weeks | +1.8±0.3 (p<0.0001) | *SAT (cm^3^):* +3% (4440 → 4570), p<0.005  *IAAT (cm^3^):* +5% (2180 → 2290), p<0.005  *TG (mmol/L):* +48% (0.99 → 1.47), p<0.05  *LDL (mmol/L):* +6% (3.2→ 3.4), p=ns  *HDL (mmol/L):* -2% (1.36 → 1.33), p<0.05  *fp-Glucose, fp-Insulin, HOMA-IR*: p=ns |
| (47) | Non-diabetic men (n=7) | One group | Isoenergetic diet + 1.5g/kg/d fruct (+18%EE) | 4 weeks | p=ns with diet | *VLDL-TAG (mmol/L):* +72% (p<0.05) within 1 week  *TAG (mmol/L): +*36% (p<0.05) within 1 week  *fp-Glucose (mmol/L):* increased at week 4  *Lipid oxidation (mg/kg/min):* trend towards a ↓ (p=0.09)  *Body composition, fp-Insulin, IMCL, total cholesterol: p=ns* compared to isoenergetic diet |
| (46) | Non-diabetic  overweight  individuals (n=20) | Randomised  Parallel  Participants blinded | 1. WM + 150g/d (600 kcal) fructose (HFrd) 2. WM + 150g/d (600 kcal) glucose (HGld) | 4 weeks | HFrD +0.2±0.6 (p=ns)  HGlD +1.7±0.4 (p=0.001)  HFrD vs HGlD, p=ns | *fp-Glucose (mmol/L):* HFrD +0.24 (p=0.014), HGlD +0.14 (p=ns), HFrD vs HGlD, p=ns  *fp-Insulin (pmol/L):* HFrD +9 (p=ns), HGlD +14 (p=0.037), HFrD vs HGlD, p=ns  *HOMA-IR:* HFrD +0.35 (p=ns), HGlD +0.59 (p=0.020), HFrD vs HGlD, p=ns  *TAG (mg/L):* HFrD +350 (p=0.004), HGlD +0 (p=ns), HFrD vs HGlD, p=0.045  *Total body fat, SAT, VAT, IMCL, LDL, HDL*: p=ns vs baseline & p=ns between groups |
| (41) | Non-diabetic individuals (n=47) | Randomised  Parallel | Standard diet + 1L/day:   1. Sucrose-sweetened cola (50% glu/50% fruct) (430 kcal/d) 2. Aspartame-sweetened diet cola (4 kcal/d) 3. Milk (454 kcal/d) 4. Water (0 kcal/d) | 6 months | No comparison with baseline  p=ns between groups | *VAT (cm^3^)*: Regular cola vs milk 31%, p<0.05; Regular cola vs diet cola/water, p=ns; comparison 4 groups ANOVA p=0.03  *Muscle fat (AU):* Comparison 4 groups ANOVA p<0.05b  ^a^*Abdominal SAT (cm^3^)*: Regular cola 4.98; diet cola -2.79; milk 3.10; water -4.30; comparison 4 groups ANOVA p=0.07  *VAT/SAAT*: Regular cola 18.1; diet cola 4.59; milk -12.5; water 3.90; comparison 4 groups ANOVA p=0.0013  *TG (mmol/L)*: Regular cola 32.7; diet cola – 0.30; milk -14.1; water -14.2; comparison 4 groups ANOVA p=0.001  *Total cholesterol (mmol/L)*: Regular cola 11.4; diet cola 0.63; milk -5.89; water -0.16; comparison 4 groups ANOVA p=0.004  *Total fat mass, fp-Glucose, fp-Insulin, HOMA-IR*: Comparison 4 groups ANOVA p=ns |

All results show mean ± SEM unless stated otherwise

^a^ Mean % change from baseline

^b^ Mean ± SD

FFM, fat free mass; EE energy excess; HFrD, high fructose diet; TAG, triacylglycerol; VLDL, very low density lipoprotein; fp, fasting plasma; HISI, hepatic insulin sensitivity index; WM, weight maintenance diet; HGlcD, high glucose diet; IMCL, intramyocellular lipid; LDL, low density lipoprotein; HDL, high density lipoprotein; EAA, essential amino acids; TG, triglycerides; HOMA-IR, Homeostatic model assessment insulin resistance; SAT, subcutaneous adipose tissue; VAT, visceral adipose tissue; PNPLA3, Patatin-like phospholipase domain-containing protein 3; BMI, body mass index; IAAT, intrabdominal adipose tissue; ANOVA, analysis of variance.

Supplementary table 2. Summary of studies examining the impact of fat over-feeding on metabolic parameters

| **Ref** | **Participants** | **Design** | **Intervention** | **Duration** | **Body weight (kg)** | **Impact on other metabolic measurements** |
| --- | --- | --- | --- | --- | --- | --- |
| Shorter duration (≤ 1 week) | | | | | | |
| (82) | Non-diabetic men (n=15) | One group | Habitual diet + 800ml cream / day) - high fat high energy (HFHE) diet (added 2632kcl/day (94% fat)) | 3 days | p=ns for BMI (no data on body weight) | *TG (mmol/L):* 1.3 → 2.9 (p<0.001)  *fp-Insulin (mu/L):* 9.1 → 21.4 (p<0.001)  *HOMA-IR*: 2.0 → 4.9 (p=0.001)  *fp-Glucose:* p=ns |
| (75) | Non-diabetic men   1. South Asian (n=10) 2. White (n= 10) | One group | High fat diet (+50% EE: 60% fat, 25% CHO, 15% protein) | 4 days |  | *HDL (mmol/L):* South Asian 1.1 → 1.3; White 1.2 → 1.4 (p=0.001)  *Insulin (μU/ml):* South Asian 11.8 → 14.3; White 10.5 → 12.7 (p=0.04)  *HOMA-IR:* South Asian 2.6 → 3.2; White 2.3 → 2.8 (p=0.04)  *Oral glucose insulin sensitivity index (ml/min/m^2^):* South Asian 433.2 → 330.1; White 473.7 → 416.8 (p=0.01)  *Total cholesterol, LDL, glucose:* p=ns  p-values show comparison before & after over-feeding  All comparisons between ethnicities for over-feeding, p=ns |
| (76) | Non-diabetic men   1. South Asian (n=10) 2. White (n=10) | One group | High fat diet (+50% EE: 60% fat, 25% CHO, 15% protein).  Subjects stayed in respiration chamber mimicking a sedentary lifestyle. | 3 days |  | *HDL (mmol/L):* South Asian 1.1 → 1.3; White 1.2 → 1.4 (p=0.001)  *Insulin (μU/ml):* South Asian 11.8 → 14.3; White 10.5 → 12.7 (p=0.04)  *HOMA-IR:* South Asian 2.6 → 3.2; White 2.3 → 2.8 (p=0.04)  *Oral glucose insulin sensitivity index (ml/min/m^2^):* South Asian 433.2 → 330.1; White 473.7 → 416.8 (p=0.01)  *Total cholesterol, LDL, glucose:* p=ns  p-values show comparison before & after over-feeding  All comparisons between ethnicities for over-feeding, p=ns |
| Longer duration (< 1 week) | | | | | | |
| (83) | Non-diabetic lean individuals (n=29) | Randomised  Cross-over | 1. High fat diet + normal protein (NP)   High fat diet + high protein (HP) | 2 weeks | p=ns vs WM  p=ns NP vs HP | *TG (mmol/l):* HP vs WM lower TGs (p=0.0007); HP vs NP (p=ns)  *Lean mass (kg):* HP vs NP higher lean mass (p=0.003)  *Fat mass (kg*): HP vs NP lower fat mass (p=0.02)  *fp-Glucose, fp-Insulin, HOMA-IR*: p=ns vs WM; p=ns NP vs HP |
| (61) | Non-diabetic individuals (n=18) | One group | 2x fast-food based meals/day (aim 5-15% weight gain)  Baseline (mean): Kcal/day 2273, CHO 48%, fat 36% (38% sat fat)  Study (mean): Kcal/day 5753, CHO 45%, fat 43% (43% sat fat) | 4 weeks | ^b^+6.4 (p<0.001) | Waist circumference (cm): 76.4 → 83.1 (p<0.001)  *Body fat (%):* 20.1 → 23.8 (p<0.001)  *HOMA-IR*: 0.89 → 1.6 (p=0.002) |
| (55) | Non-diabetic normal weight individuals (n=39) | Randomised  Parallel  double-blind, (LIPOGAIN) | Standard diet +   1. Muffins high in SFAs (palm oil) 2. Muffins high in n-6 PUFAs (sunflower oil)   Quantity adjusted for 3% weight gain  Muffins matched for energy, fat, protein, CHO, cholesterol. | 7 weeks | ^b^SFA +1.6±0.96  PUFA +1.6±0.85  SFA vs PUFA, p=ns | *VAT (L):* SFA 0.22, PUFA 0.11 (p=0.035)  *Abdominal SAT (L):* SFA 0.34, PUFA 0.25 (p=ns)  *VAT/SAT ratio:* SFA 0.01, PUFA 0.00 (p= 0.073)  *Total body fat (L):* SAT 1.5, PUFA 0.97 (p=0.013)  *Lean tissue (L):* SFA 0.31, PUFA 0.86 (p=0.015)  *fp-Glucose, fp-Insulin, HOMA-IR, pancreatic fat:* SFA vs PUFA, p=ns |
| (84) | Non-diabetic  individuals (n=29) | One group | +40% EE: 41% CHO, 44% fat (40% SFAs), 15% protein | 8 weeks | +7.6±2.1 | *Body fat (%):* 19.4 → 22.2 (p<0.01)  *Abdominal SAT (kg):* 4.1 → 5.4 (p<0.01)  *VAT (kg):* 0.58 → 0.94 (p<0.01)  *Cholesterol (mg/dl):*171 → 196 (p<0.01)  *LDL (mg/dl):99* → 120 (p<0.01)  *fp-Insulin* (mU/ml): 5.4 → 8.3 (p<0.05)  *Insulin sensitivity (mU/min . m^2^ insulin*)*:* 2.87 → 2.35 (p<0.001)  *HDL, TG, fp-Glucose , IMCL:* p=ns |
| (57) | Non-diabetic overweight individuals  (n=60) | Randomised  Parallel  double-blind, (LIPOGAIN-2) | Standard diet +   1. Muffins high in SFAs (palm oil) 2. Muffins high in n-6 PUFAs (sunflower oil)   Quantity adjusted for 3% weight gain  Muffins matched for energy, fat, protein, CHO, cholesterol | 8 weeks | ^b^SFA +2.31±1.38  PUFA +2.01±1.90  SFA vs PUFA, p=ns | *VAT (L):* SFA 0.37, PUFA 0.26 (p=ns)  *Total body fat (L):* SAT 2.22, PUFA 1.77 (p=ns)  *Total cholesterol, HDL, LDL:* Profiles worse in SFA group, p<0.05  *fp-Glucose, fp-Insulin, HOMA-IR, pancreatic fat:* SFA vs PUFA, p=ns |

All results show mean ± SEM unless stated otherwise

^a^ Mean % change from baseline

^b^ Mean ± SD

BMI, body mass index; TG, triglycerides; fp, fasting plasma; HOMA-IR, Homeostatic model assessment insulin resistance; EE energy excess; CHO, carbohydrate; HDL, high density lipoprotein; LDL, low density lipoprotein; WM; weight maintenance diet; SFA, saturated fatty acids; n-6 PUFA, omega 6 polyunsaturated fatty acids; VAT, visceral adipose tissue; SAT, subcutaneous adipose tissue; IMCL, intramyocellular lipid.

Supplementary table 3. Summary of studies examining the impact of carbohydrate and fat over-feeding on other parameters

| **Ref** | **Participants** | **Design** | **Intervention** | **Duration** | **Body weight (kg)** | **Impact on other metabolic measurements** |
| --- | --- | --- | --- | --- | --- | --- |
| Shorter duration (≤ 1 week) | | | | | | |
| (44) | Non-diabetic individuals  (n=12) | Randomised  Cross-over | Hypercaloric diet (+45% EE) + high in sucrose   1. Low protein, high fat hypercaloric diet (LP-HF): 5% protein, 25% fat 2. High protein, low fat hypercaloric diet (HP-LF): 20% protein, 10% fat | 6 days  4-8 week washout period | LP-HF +0.7±0.1 (p=ns)  HP-LF +1.4±0.2 (p<0.01) | *IMCL (mmol/kg ww)*:  LP-HF: 7.1 → 8.8  HP-LF: 6.2 → 6.9  Comparison between groups, p<0.002 |
| (45) | Non-diabetic men (n=37) | Randomised  Parallel | 1. HFrD (+3.5g fructose/kg/FFM, +35% energy) 2. High fat diet (Fat) (+30% energy as fat) 3. High-fructose high-fat diet (FruFat) (3.5g fructose/kg/FFM +30% energy as fat) | 7 days | p=ns | *VLDL TAG (mmol/L): Fru 0.55* → 0.87 (p<0.05); Fat 0.58 → 0.45 (p<0.05); FruFat 0.51 → 0.55 (p=ns)  *fp-Insulin (mU/L): Fru 8.7* → 9.6; Fat 8.3 → 8.8; FruFat 8.8 → 11.1  *fp-Glucose*: p=ns compared to controls and between groups |
| (85) | Non-diabetic men (n=55) | Randomised  Parallel | WM diet + either:   1. 1.5/3/4g fructose/kg/FFM (F1.5, F3, F4) 2. 3g/kg/day glucose (G3.0) 3. 30% energy as SFAs (Fat30%) | 6-7 days | p=ns all group vs controls | *Insulin (mU/L):* F3 9.4 → 12.5 (p<0.01); all other groups p=ns vs controls  *HISI*: F3 6.1 → 4.8 (p<0.01); F4 5.6 → 4.4 (p<0.05); all other groups p=ns vs controls  *Hepatic glucose production* *(mg/kg/min):* F4 1.81 → 2.11 (p<0.01); G3.0 2.16 → 2.30 (p<0.01); all other groups p=ns vs controls  *Glucose:* p=ns all group vs controls |
| Longer duration (> 1 week) | | | | | | |
| (42) | Non-diabetic lean men (n=36) | Randomised  Parallel | Hypercaloric diet (+40% EE)   1. High fat high sugar + ↑ meal size (HFHS-S) 2. High fat high sugar + ↑ meal frequency (HFHS-F) 3. High sugar + ↑ meal size (HS-S) 4. High sugar + ↑ meal frequency (HS-F)   Controls: ad libitum diet | 6 weeks | *BMI (kg/m^2^):* HFHS-S +0.6 (p<0.05)  HFHS-F +0.9 (p<0.01)  HS-S +0.8 (p<0.001)  HS-F + 0.5 (p=ns)  (no data on body weight) | *IAAT (L):* HFHS-S 0.45 → 0.45 (p=ns); HFHS-F 0.53 → 0.59 (p=0.004); HS-S 0.39 → 0.44 (p=ns); HS-F 0.50 → 0.55 (p=ns)  *SAT (L):* HFHS-S 0.25 → 0.23 (p=ns); HFHS-F 0.29 → 0.33 (p=0.007); HS-S 0.19 → 0.22 (p=ns); HS-F 0.26 → 0.29 (p=0.020)  *TG (mmol/L):* HFHS-S 0.69 → 0.78 (p=ns); HFHS-F 0.56 → 0.84 (p=0.012); HS-S 0.66 → 0.83 (p=ns); HS-F 0.68 → 0.85 (p=ns)  *VAT, fp-Glucose, fp-Insulin*: p=ns from baseline for all groups |
| (26) | Non-diabetic individuals (n=38) | Randomised  Parallel | Hypercaloric diet (1,000 excess kcal/day)   1. SAT: Mainly SFAs (76% SFAs, 21% MUFAs, 3% PUFAs) 2. UNSAT: Mainly USFAs (57% MUFAs, 22% PUFAs, 21% SFAs) 3. CARB: 100% simple sugars | 3 weeks | p=ns | *fp-Insulin (mU/L):* SAT 8.1 → 9.5 (p<0.05), UNSAT 9.1 → 9.5 (p=ns), CARB 10.3 → 11.14 (p=ns)  *HOMA-IR:* SAT 1.9 → 2.2 (p<0.05), UNSAT p=ns, CARB p=ns  *LDL* *(mmol/L):* SAT +0.3 (p<0.01), UNSAT p=ns, CARB p=ns  *HDL (mmol/L):* SAT +0.3 (p<0.01), UNSAT p=ns, CARB p=ns  *Lipolysis (whole-body glycerol Ra during euglycemic hyperinsulinemia) (mmol/kg . min):* SAT 2.08 → 2.31 (p<0.05), UNSAT 2.59 → 2.14 (p<0.05), CARB 2.15 → 2.27 (p=ns)  *VAT, SAT, TG*: all p=ns |

All results show mean ± SEM unless stated otherwise

^a^ Mean % change from baseline

^b^ Mean ± SD

HFrD, high fructose diet; FFM, fat free mass; VLDL, very low density lipoprotein; TAG, triacylglycerol; fp, fasting plasma; WM, weight maintenance diet; HISI, hepatic insulin sensitivity index; EE energy excess; BMI, body mass index; IAAT, intrabdominal adipose tissue; SAT, subcutaneous adipose; TG, triglyceride; VAT, visceral adipose tissue; SFA, saturated fatty acids; MUFA, monounsaturated fatty acids; PUFA, polyunsaturated fatty acids; HOMA-IR, Homeostatic model assessment insulin resistance; LDL, low density lipoprotein; HDL, high density lipoprotein; IMCL, intramyocellular lipid.
